# The effect of P2RX7 functional SNPs on osteoblast cell phenotype, function and signalling- roles in bone homeostasis and osteogenesis

**DOI:** 10.1007/s11302-026-10171-5

**Published:** 2026-06-27

**Authors:** Luke Tattersall, Qiguang Wang, Anna Lisa Giuliani, Elena Adinolfi, Alison Gartland

**Affiliations:** 1 School of Medicine and Population Health, Division of Clinical Medicine, The Mellanby Centre for Musculoskeletal Research, The University of Sheffield, Sheffield, UK; 2 College of Biomedical Engineering, National Engineering Research Center for Biomaterials, Sichuan University, Chengdu, 610065 Sichuan China; 3 Department of Medical Sciences, Section of Experimental Medicine, University of Ferrara, Ferrara, Italy

**Keywords:** ATP, Bone, Osteoblasts, P2RX7, Purinergic Signalling, SNP, His155Tyr, Arg307Gln, Glu496Ala

## Abstract

Bone cells are known to express multiple purinergic receptors. These are involved in various aspects of bone physiology depending on the cell type and receptor involved, both in normal and disease states, with extracellular ATP being particularly abundant in the bone microenvironment. The P2X7R subtype has been the subject of in-depth research in bone homeostasis. Different splice isoforms and single nucleotide polymorphisms (SNPs) have been discovered in the *P2RX7* gene, these have functional implications on the receptor, conferring both gain of function (GOF) and loss of function (LOF), with consequences for bone phenotype, turnover and mass. We examined the full length *P2RX7A*, truncated *P2RX7B,* their co-expression and different receptor SNPs. The SNPs included the c.489C > T variant (p.His155Tyr) GOF, c.946G > A (p.Arg307Gln) LOF and c.1513A > C (p.Glu496Ala) LOF. The effect of these isoforms and SNPs on the normal cellular behaviour of the TE85 osteoblastic cell line were measured for intracellular calcium uptake, pore formation, cell proliferation, Alkaline phosphatase (ALP) activity, mineralisation, and changes in osteogenic gene expression. The results demonstrate that transfection of *P2RX7* isoforms and SNP variants influenced several important bone processes. Compared to TE85 cells all isoform variants had an increased calcium response with only the co-transfected *P2RX7AB* having pore formation. This variant also had the highest mineralisation and ALP activity but was the least proliferative. Of the co-transfected SNP variants only the TE85 + P2RX7B + 155Y GOF cell line had both pore formation and an increased calcium response. Mineralisation was lower in all SNP cell lines as was ALP activity, however, proliferation was increased. Mechanistically, gene expression arrays showed that Nuclear Factor of Activated T-cells 1 (*NFATc1*) increased in the highly proliferative cell variants. Osteogenic genes such as Bone morphogenetic protein 2 (*BMP2)*, *ALP* and collagen type I alpha 1 chain (*COL1A1*) were increased in the cell variants that had high mineralisation and ALP activity, and were decreased in the highly proliferative variants, demonstrating multiple bone phenotypes depending on GOF or LOF. In conclusion, this study extends our knowledge into the role of the P2X7R in maintaining bone homeostasis and provides assessment of *P2RX7* polymorphisms in osteoblasts. In future this could help to identify associations between specific variants and increased/reduced bone mineral density or accelerated bone loss/fracture risk in a number of bone related conditions.

**Supplementary Information:**

The online version contains supplementary material available at 10.1007/s11302-026-10171-5.

## Introduction

Osteoblasts are a major component of the skeletal system involved in the process of bone formation and remodelling [1], they are responsible for the synthesis and formation of bone matrix proteins and mineral [2]. They derive from mesenchymal origin which undergo lineage commitment, differentiating to progenitor, immature and finally mature osteoblast cells [3]. These differentiated cells counteract the bone resorption effects of osteoclasts throughout the lifelong process of skeletal remodelling. This is a tightly regulated process involving transcription factors, cytokines and growth factors [4]. Disruption to osteoblast function may cause excessive or insufficient production of bone matrix resulting in pathologies such as osteoporosis and osteoarthritis [5], or it could contribute towards a cancerous phenotype such as in osteosarcoma bone cancer, where high levels of ectopic bone are produced [6].

Purinergic signalling involves extracellular nucleotides signalling via P2 receptors [7]. These are subdivided into P2Y GPCRs with eight subtypes and P2X ligand gated ion channels with seven subtypes [8]. Of the P2X receptors the P2X7R is the largest of the P2X family with 595 amino acids, the gene is located on chromosome 12q24.31 [9], it is unique in that it requires higher levels of ATP for activation and can act as a cation channel or form a large non selective pore permeable to molecules of up to 900 Da [10]. The full length receptor is known as P2X7RA with eleven different naturally occurring splice variants (P2X7RA-L) [11–14]. P2X7RB, P2X7RE, P2X7RG and P2X7RJ, lack the extended C-terminal tail typical of P2X7RA, with P2X7RB unique as it remains a functional ion channel, although unable to form the large conductance pore [13]. In addition to this the *P2RX7* gene has several known polymorphisms. The c.489C > T variant (p.His155Tyr) was the first identified GOF SNP [15]. This SNP is located on exon 5 at position 489 (cytosine to thymine) [16] and transforms amino acid 155 from histidine to tyrosine in the receptorʼs extracellular region. This SNP demonstrated a rise in calcium influx in both primary lymphocytes [15] and human embryonic kidney cells [17], and has been shown to form a pore in further studies [16, 18, 19]. The LOF c.946G > A (p.Arg307Gln) SNP is located on exon 9 [16], at residue 307, this SNP converts arginine to glutamine which results in a lack of both channel and pore function [20]. A further LOF c.1513A > C (p.Glu496Ala) SNP is located on exon 13 [16] at residue 496, in the C-terminal of the receptor. This SNP converts glutamic acid to alanine and prevents pore formation, but retains some channel function [21, 22]. P2X7R and its associated splice variants have been studied in bone cell function [23], and in bone cancer [11, 24, 25]. In bone cells they modulate cellular processes such as differentiation, proliferation, apoptosis, ATP release and mineralisation [23], therefore affecting both resorption and bone formation, however, the effects of these SNPs on these processes are unknown. Understanding these genetic influences can lead to better insights into bone diseases and inform the development of targeted therapies.

Previous association studies have correlated various SNPs with clinical phenotypes. In a cohort of 1764 postmenopausal women the LOF p.Glu496Ala was correlated with an increased fracture risk over 10 years [26]. . Building on this, in a previous study by our group, we genotyped 506 postmenopausal women for P2RX7 SNPs and examined their lumbar spine bone mineral density (BMD at baseline and at a 5–6 year follow up. We found an association in the c.946G > A (p.Arg307Gln) with low BMD in the lumbar spine, both at the initial baseline and at the follow up visit [27]. Supporting the implication of p.Arg307Gln and its effect on bone loss in postmenopausal women. A further study in 1694 Danish postmenopausal women genotyped for P2RX7 SNPs demonstrated that for BMD there was 40% more bone loss at the hip (assessed at baseline and after 10 years) in women with the p.Arg307Gln variant, compared with women who were wild type at this position. Interestingly, the GOF p.Ala348Thr polymorphism was associated with a lower vertebral fracture incidence 10 years after menopause suggesting *P2RX7* expression and function is critical to preventing bone loss [28].

An association between the risk of stress fracture injury and the LOF SNP p.Glu496Ala was reported in 210 active duty Israeli soldiers and 518 elite athletes from the UK and the US. Additionally, a stress fracture injury reduction was linked to a *P2RX7* GOF SNP p.Ala348Thr [29].

Finally, in a study of Dutch fracture patients BMD was assessed at the total hip, lumbar spine, and femoral neck after genotyping 690 women and 231 men aged 50 years for 15 non-synonymous *P2RX7* SNPs. BMD was correlated with four non-synonymous SNPs (the p.Ala348Thr and p.Gln460Arg GOF and p.Gly150Arg and p.Glu496Ala LOF). BMD values at the lumbar spine increased in correlation with the p.Ala348Thr GOF polymorphism and in the male patients the p.Gln460Arg GOF polymorphism had a decrease in the risk of a lower BMD T-score value and overall risk of osteoporosis. In the cohort with the p.Glu496Ala and p.Gly150Arg polymorphism lower hip BMD values were observed [30].

As there are clear implications between P2X7R function and bone homeostasis, in this study we focused on the influence at the cellular level of the full length P2X7RA, truncated P2X7RB, co-transfected P2X7RAB and the P2X7R SNPs introduced into the A isoform of the AB variant: c.489C > T variant (p.His155Tyr) GOF, c.946G > A (p.Arg307Gln) LOF and c.1513A > C (p.Glu496Ala) LOF , to determine the effects on osteoblast function and signalling. The P2X7R exists as a functional complex that can include both the full-length P2X7RA isoform and the truncated P2X7RB isoform, these isoforms can co-assemble and modulate each otherʼs activity with important implications for physiological processes. Keeping the truncated P2X7RB variant and introducing SNPs specifically in the P2X7RA variant after co-transfecting both allows us to investigate how the P2X7RB influences the functional impact of these SNPs in P2X7RA. Since the P2X7RB can modulate the activity of the P2X7RA, studying them together reflects a more physiologically relevant scenario. This approach will help to reveal whether the P2X7RB variant can compensate for or exacerbate the functional changes induced by SNPs in the P2X7RA variant, thus providing a clearer understanding of the receptorʼs role in bone-related conditions.

In this study, we report the effect of these variants and three *P2RX7* SNPs in osteoblast functions, including intracellular calcium uptake, pore formation, cell proliferation, ALP activity, mineralisation, and changes in osteogenic gene expression. These effects help explain the reduced BMD, accelerated bone loss, and increased bone fracture risk observed in patients with *P2RX7* SNPs. This study also extends our knowledge into the functional role of the P2X7R in maintaining bone homeostasis, it may in the future help identify individuals with increased/reduced BMD or accelerated bone loss or fracture risk.

## Materials and methods

### Cell culture and transfections

The human OS cell line HOS-TE85 (referred to as TE85) were kindly provided from Professor Jim Gallagher (Liverpool, UK). Cell lines were maintained in DMEM© + Glutamax™ (Life Technologies, UK) medium containing 10% FBS with 100 units/mL penicillin and 100μg/mL streptomycin antibiotics (Life Technologies, UK) (referred to as complete medium). For transfections, cDNAs containing the full-length human *P2RX7A* or the truncated human *P2RX7B* splice variant, were previously generated by insertion of their sequence into a mammalian expression vector pcDNA 3.1. For the *P2RX7A* the plasmid carried gentamicin resistance, and for *P2RX7B* the plasmid carried hygromycin B resistance. Stable TE85 expressing *P2RX7A*, *P2RX7B* and *P2RX7AB* cell lines were obtained by transfection of the cDNAs into TE85 cells using lipofectamine LTX (Life Technologies, UK), followed by antibiotic selection with either G418 sulphate, hygromycin or both using 0.2–0.8mg/ml and have been used in our previous studies [11, 24] The parental TE85 naïve cells contained an empty vector construct and were used as the baseline control for comparison with receptor variants.

For co-transfections with SNPs, GFP-tagged cDNAs containing LOF polymorphisms c.946G > A (p.Arg307Gln) c.1513A > C (p.Glu496Ala) and GOF polymorphism c.489C > T variant (p.His155Tyr) were kindly gifted from Prof Wiley (University of Sydney at Nepean Hospital, Australia). These were transfected in the same manner into the TE85 variant already expressing *P2RX7B*. The following cell lines were therefore used in this study: TE85, TE85 + P2X7RA, TE85 + P2X7RB, TE85 + P2X7RAB and SNP variants: GOF TE85 + P2X7RB + 155Y, LOF TE85 + P2X7RB + 307Q and TE85 + P2X7RB + 496A. All cells were grown at 37 °C in a humidified 5% CO_2_ incubator. All cells were routinely mycoplasma tested to ensure experiments were carried out with mycoplasma free cells.

### RNA extraction and cDNA synthesis

RNA was extracted from cells using the ReliaPrep™ RNA Miniprep System kit in accordance with the manufacturerʼs protocol (Promega, UK). RNA was reverse transcribed using the Applied Biosystems™ high capacity RNA to cDNA™ Kit in accordance with the manufacturerʼs protocol. End point PCR primers were designed using Pubmed gene sequences. RNA yield was determined by nanodrop® spectrophotometer ND-1000. RNA was reverse transcribed using ImProm-II™, using 1µg/µL of the RNA samples, these were mixed with 1µL of the 0.5µg/µL random primers and heated to 75°C for 1 min with cooling on ice. The reverse transcription mix was then added which included ImProm-II™ 5X reaction buffer, ImProm-II™ reverse transcriptase, 3mM MgCl_2_, 0.5mM of each dNTP and nuclease free water. Samples were gently mixed and incubated at room temperature for 10 min, 40°C for 50 min and finally 75°C for 10 min. Samples were then cooled on ice and stored at 4°C.

### Primer design to cover regions containing P2RX7 SNPs

Primers spanning non-synonymous SNPs within *P2RX7* were designed as shown in Table 1.

**Table 1:** Forward and reverse primers spanning non-synonymous SNPs within the *P2RX7* and their corresponding product lengths

SNP variant	Forward	Reverse	Product length
155Y SNP: c.489C > T variant (p.His155Tyr):	TTGTGTCCCGAGTATCCCAC	TCAATGCCCATTATTCCGCC	413
307Q SNP: c.946G > A (p.Arg307Gln):	CACTGCCGTCCCAAATACAG	TGGACTCGCACTTCTTCCTG	370
496A SNP: c.1513A > C (p.Glu496Ala):	ACCAGAGGAGATACAGCTGC	TACTGCCCTTCACTCTTCGG	399

### End point PCR

The cDNA was amplified using Promega GoTaq Flexi DNA polymerase. The reaction mix was as follows: DNA Polymerase (5µ/µL), 5X GoTaq**®** Reaction buffer, dNTPs (0.2mM), MgCl_2_ (1.5mM) upstream and downstream primer (0.5µM) and template cDNA (1µg). The PCR samples were initially denatured for 2 min at 95°C for one cycle, followed by 35 cycles of denaturation at 90°C for 30 s, annealing at primer TM for 30 s, and extension at 72°C for 30 s. A final extension was then performed at 72°C for 5 min for one cycle before samples were held indefinitely at 4°C.

### Sequencing

DNA sequencing was performed by the University of Sheffield Core Genomics Facility using an Applied Biosystemsʼ 3730 DNA Analyser with BIgDye Terminator v3.1 cycle sequencing kit. PCR products were treated to remove excess primers and dNTPs using the ExoSAP protocol.

### Intracellular calcium assay

Intracellular calcium intake was measured using the Invitrogen Fluo-4 Direct Calcium Assay Kits according to the manufacturerʼs instructions (Life Technology, UK). In brief, 10,000 cells were resuspended into 96-well plates with HBSS loaded with Fluo-4 Direct reagents for 15 min at 37 °C and placed into a SpectraMax M5e Microplate Reader (Molecular Devices, Sunnyvale, CA). Cells were then stimulated by auto-injection of 200μM BzATP (2′(3′)-O-(4-benzoylbenzoyl)adenosine-5′-triphosphate), and fluorescence measured (488/530nm excitation/emission), indicating intracellular calcium intake; from 100 s before, to 10 min after the addition of BzATP.

### Measurement of plasma membrane permeability (pore formation)

Increases in plasma membrane permeability were measured by monitoring ethidium bromide up-take. Briefly, 10,000 cells were seeded into 96-well plates and incubated at 37 °C with 100μL Hankʼs Balanced Salt Solution (HBSS) (ThermoFisher Scientific UK) media containing ethidium bromide to a final concentration of 100μM with/without 300μM BzATP (previously shown to activate pore formation in our previous studies [24]). Induction of pore formation was detected at 360 nm excitation and 580 nm emission for 90 min, with readings taken every 5 min using a SpectraMax M5e Microplate Reader (Molecular Devices, Sunnyvale, CA).

### Measurement of cell proliferation

Cell proliferation was measured using the CellTiter 96® AQueous Non-Radioactive Cell Proliferation Assay according to the manufacturerʼs instructions (Promega, Southampton, UK). Cells were seeded into 96-well plates at 1,000 cells per well in 100µL of complete medium. Cell proliferation at day 4 was measured by the absorbance of the formazan product produced by the cells, the plate was read at 490nm using a SpectraMax M5e Microplate Reader (Molecular Devices, Sunnyvale, CA).

### ALP activity

All cell variants were seeded into 96-well plates at a density of 1,000 cells per well in 100µL of complete medium with a range of BzATP concentrations (0, 1, 10, 100µM). After 3-days of culture, cells were washed with PBS and lysed in nuclease-free water with 1M Tris pH 8.0–8.3, 0.2M MgCl_2_ and 0.2% Triton-X. Cell lysates were obtained after three freeze and thaw cycles. ALP activity was measured by using pNPP (Sigma, Poole, UK) as the chromogenic ALP substrate in the presence of Mg2^+^ ions in a buffered solution. The absorbance was read at 405nm using a SpectraMax M5e Microplate Reader. To normalise the ALP activity per cell, dsDNA (double-stranded DNA) concentration was quantified using a Quant-iT™ PicoGreen dsDNA Assay Kit (Invitrogen, Paisley, UK) according to the manufacturerʼs instructions. The fluorescence (480/520nm excitation/emission) was measured using a SpectraMax M5e Microplate Reader, and fluorescence was read against a standard curve of calf thymus DNA to determine DNA content. ALP activity was finally normalised to dsDNA content.

### Mineralisation

TE85 variant cells were seeded into 24-well plates at 50,000 cells/well in 1mL complete medium and cultured until confluent. The medium was then replaced with osteogenic medium (Complete DMEM with 0.5% FBS, 50μg/mL L-Ascorbic acid and 10nM Dexamethasone), with a range of BzATP concentrations (0, 1, 10, 100µM). The experiment then continued for a total of 7 days in osteogenic medium. Three days prior to the end of the experiment, inorganic phosphate (at a final concentration of 0.5mM, pH 4.2) was added to each well. After this period wells were washed once in PBS, fixed in 100% ethanol, rinsed in PBS and incubated in 40mM Alizarin red (pH 4.2; Sigma, Poole, UK) for 1 h at room temperature. The cells were then washed in 95% ethanol and air-dried overnight. The plates were scanned on a high-resolution flat-bed scanner. The percentage of each well stained with Alizarin red was quantified using Image J software (NIH: http://rsb.info.nih.gov/ij/).

### Real-time PCR

Expression of a series of selected genes (Supplementary Table 1) were determined by real-time PCR using the TaqMan® Assays in 384-Well Microfluidic Cards in triplicate in a 7900HT Fast Real-Time PCR system according to the manufacturerʼs instructions (Applied Biosystems). In brief, 50μl cDNA sample (equivalent to 1 μg cDNA) was mixed with 50μl Taqman Universal PCR Master Mix (2X) and loaded into each filling reservoir of the Taqman microfluidic cards. The cards were then centrifuged twice for 1 min at 280g. The cards were placed in the microfluidic card specific block and the thermal cycling conditions were 2 min at 50˚C and 10 min at 94.5˚C, followed by 40 cycles of 30 s at 97˚C and 1 min at 59.7˚C. The assay for each gene on the microfluidic card was carried out in duplicate, based on our customised design. The calculation of the threshold cycle (Ct) values was performed using the SDS 2.2 software and RQ Manager 1.2.1 (Applied Biosystems), using the automatic settings of the baseline and the threshold. A comparative CT experiment was run to allow determination of the change of expression (fold increase) of the target cDNA in the test sample relative to the TE85 reference sample.

### Statistical analysis

Unless otherwise stated, data shown are mean ± SD. Data set was first tested for normality using the DʼAgostino-Pearson omnibus test and the Shapiro–Wilk test. Only the data that passed both normality tests were analysed by One-way ANOVA test; otherwise a non-parametric analysis was employed. Level of significance was calculated using Graphpad Prism (GraphPad Software Inc, San Diego, CA, USA) where * = *p* < 0.05, ** = *p* < 0.01, *** = *p* < 0.001, **** = *p* < 0.0001.

## Results

### P2RX7 variant expression and SNP Sequencing

**Fig. 1:**
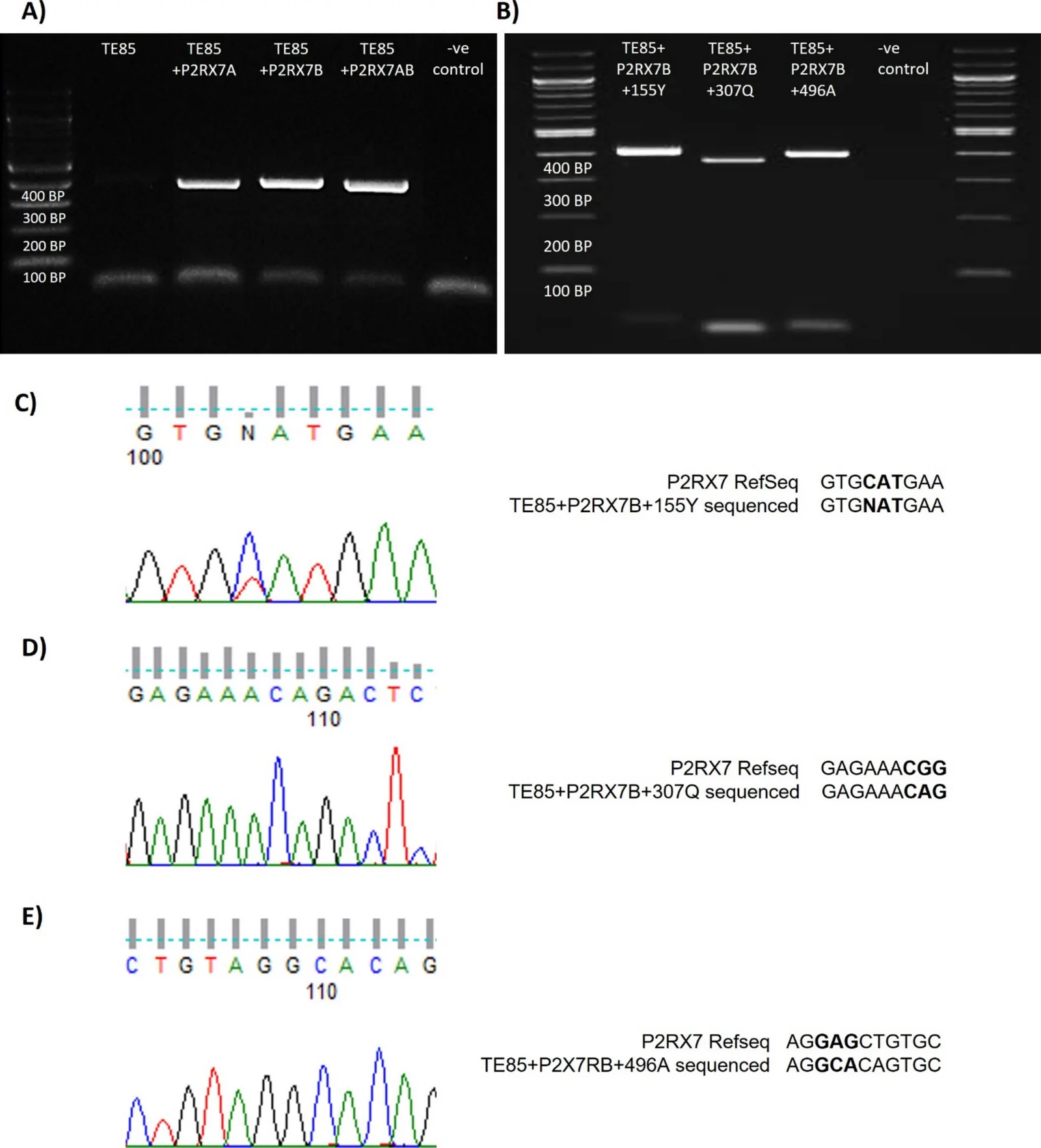
Confirmation of the transfection of P2X7R variants and sequencing of SNPs. **A**) End point PCR showing expression of the *P2RX7A*, *P2RX7B* and *P2RX7AB* genes using primers that bind in the N- terminal region product length 413 BP. **B**) Primers spanning the SNP regions were used to demonstrate expression and are showing: TE85 + P2X7RB + 155Y, product length 413BP. TE85 + P2X7RB + 307Q product length 370BP and P2X7RB + 496A product length 399BP. These products were then sequenced. **C**) CAT/TAT mixed population resulting in c.489C > T variant (p.His155Tyr) GOF. **D**) CGG to CAG resulting in c.946G > A (p.Arg307Gln) LOF. **E**) GAG to GCA resulting in c.1513A > C (p.Glu496Ala) LOF

For End point PCR performed on TE85, TE85 + P2X7RA, TE85 + P2X7RB and TE85 + P2X7RAB the primer pairing that spanned the 155Y SNP was used (product length 413 BP) as this detected a region towards the N-terminal and therefore will detect both *P2RX7A* and *P2RX7B*. The results demonstrated successful transfection of these variants into TE85 cells with bands at the expected product lengths (Fig. 1A). End point PCR was further performed on the TE85 P2X7RB + 155Y GOF cell line using the P2X7R primers spanning the 155Y SNP. The TE85 P2X7RB + 307Q LOF cell line using the P2X7R primers spanning the 307Q SNP and the TE85 P2X7RB + 496A LOF cell line using the P2X7R primers spanning the 496A SNP. As expected, all yielded a band of the correct size (Fig. 1B).

Sequencing of the PCR product from the TE85 + P2X7RB + 155Y cells identified the GOF SNP at position 155. However, peaks were present for both C and T meaning that some cells have the GOF non-synonymous SNP coding for tyrosine (TAT) but some have the wild type histidine amino acid (CAT) and therefore was a mixed cell population (Fig. 1C). Sequencing of the PCR product from the TE85 + P2X7RB + 307Q cells identified a base change from the expected CGG coding for arginine to CAG coding for glutamine (the expected non-synonymous SNP, Fig. 1D). Sequencing of the PCR product from the TE85 + P2X7RB + 496A cells identified a change to GAG coding for glutamic acid to be changed to GCA coding for alanine (the expected non-synonymous SNP, Fig. 1E). Therefore, each individual SNP was confirmed.

### P2X7R variant and SNP characteristics

**Fig. 2:**
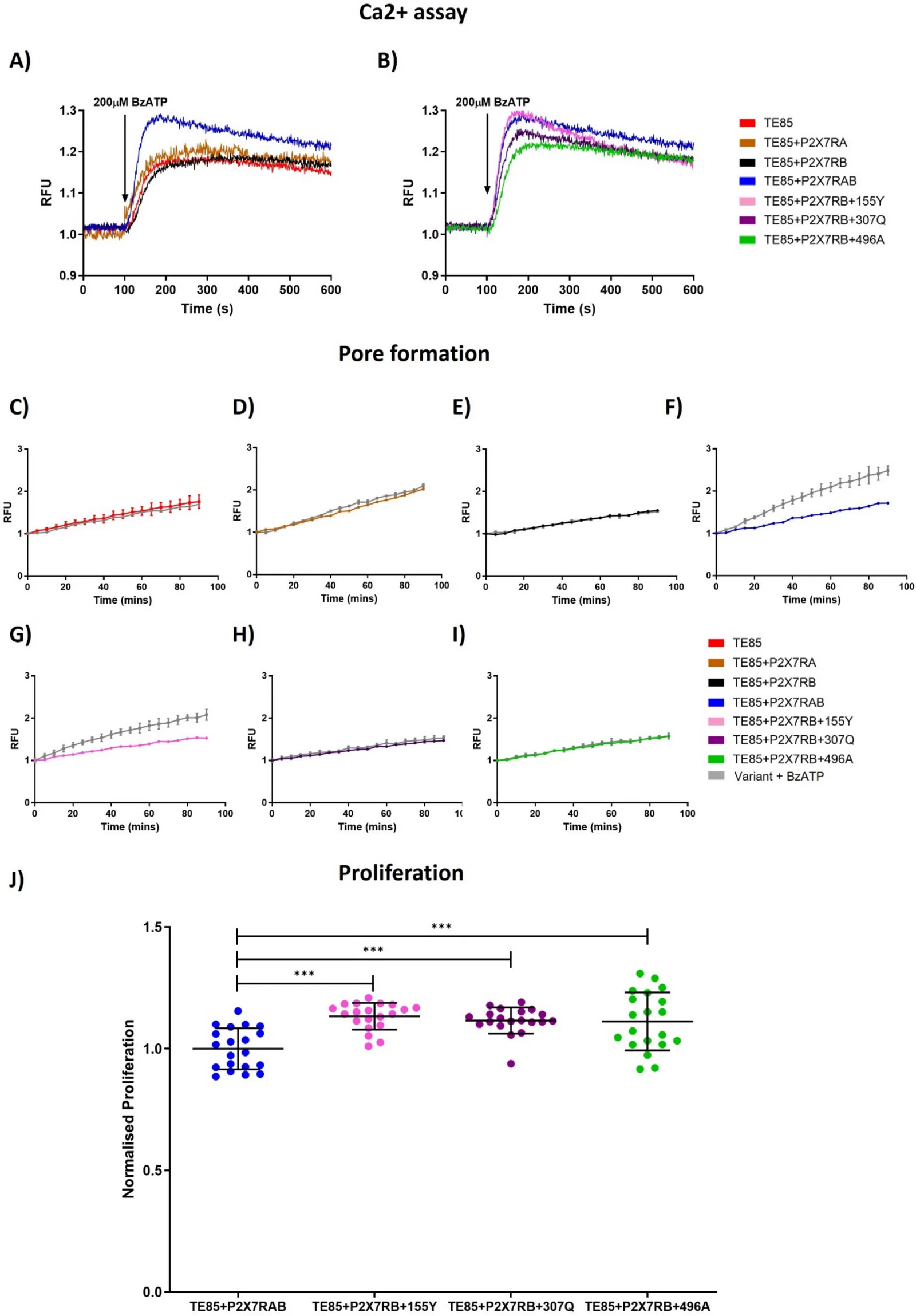
Calcium assays were assessed in all TE85 variants and SNP cell lines by loading 10,000 cells with HBSS and Fluo-4 Direct™ calcium reagent, these were then left to incubate for 15 min at 37°C prior to stimulation with 200µM BzATP. The response was detected at excitation ratio and emission wavelength 488nm and 530nm respectively from 100 s before to 10 min after stimulation with BzATP. **A)** Relative fluorescent unit (RFU) changes relative to TE85 cells for P2X7RA, P2X7RB and P2X7RAB **B)** RFU changes relative to TE85 + P2X7RAB cells for the GOF and LOF variants. Measurement of plasma membrane permeability was performed using ethidium bromide uptake, 10,000 cells were seeded into 96-well plates and incubated at 37°C with 100μL HBSS media containing ethidium bromide to a final concentration of 100μM with/without 300μM BzATP. Induction of pore formation was detected at 360nm excitation and 580nm emission for 90 min, with readings taken every 5 min. **C)** TE85 **D)** TE85 + P2X7RA **E)** TE85 + P2X7RB **F)** TE85 + P2X7RAB **G)** TE85 + P2X7RB + 155Y **H)** TE85 + P2X7RB + 307Q **I)** TE85 + P2X7RB + 496A. For proliferation 1,000 cells were seeded into a 96-well plates in complete medium, after 4 days the absorbance was read at 490nm and values normalised to day 0. **J)** Proliferation rate of the SNP variants compared to TE85 + P2X7RAB, * *p* < 0.05, ** *p* < 0.01, *** *p* < 0.001. Control data shown across panels were derived from the same experiment and are presented separately for clarity and biologically relevant comparisons

To investigate the effect of the transfection of P2X7R variants and their SNPs on the cation-channel functionality of P2X7R, cells were stimulated with 200µM BzATP and intracellular calcium uptake was measured using the Invitrogen Fluo-4 Direct Calcium Assay. The results for the TE85, TE85 + P2X7RA, TE85 + P2X7RB and TE85 + P2X7RAB demonstrated consistency with our previously published results [11] where calcium uptake levels in the P2XR7AB expressing cells was the highest, followed by P2X7RA and then P2X7RB expression cells respectively (Fig. 2A and B). For the cell line with the GOF P2X7R 155Y SNP the intracellular calcium levels were similar to the P2X7RAB variant whereas the LOF SNPs both demonstrated reduced levels (Fig. 2B). Sustained stimulation of the P2X7R with its agonist ATP enables the P2X7R to form a large irreversible pore permeable to molecules with a molecular weight of up to 900 Daltons (Da) [10]. This includes large organic dyes such as ethidium bromide (molecular weight 394). This function is unique to the P2X7R subset of purinergic receptors and has a variety of roles, therefore, this function was assessed in TE85 cells transfected with the different variants and SNPs, the using BzATP at a concentration consistent with our published studies [11, 24]. The assay demonstrated no functional pore formation ability in the TE85 cells (as previously shown [11, 24], Fig. 2C). This was similarly the case with TE85 + P2X7RA (Fig. 2D) and TE85 + P2X7RB (Fig. 2E) despite expressing P2RX7 mRNA. Only co-transfection of both A and B variants demonstrated the ability to produce a fully functional pore typical of the classical P2X7R (Fig. 2F). For SNP variants the TE85 + P2X7RB + 155Y GOF cell line produced a fully functional pore formation ability (Fig. 2G), in contrast to this no pore formation was demonstrated in the TE85 + P2X7RB + 307Q (Fig. 2H) or TE85 + P2X7RB + 496A (Fig. 2I) LOF cell lines.

P2X7Rs are known to increase and decrease cell proliferation depending on the model, cell type and isoform. We have previously shown that expression of P2X7RA, P2X7RB, and P2X7RAB increases cell proliferation in these cells [11, 24]. Therefore, in this study, we assessed cell proliferation of the SNP variants, TE85 + P2X7RB + 155Y, TE85 + P2X7RB + 307Q and TE85 + P2X7RB + 496A compared with the TE85 + P2X7RAB over four days. TE85 + P2X7RB + 155Y had the largest increase in rate of 13.3%, (*p* < 0.0001), with TE85 + P2X7RB + 307Q increasing by 11.16%, (*p* < 0.0001), and TE85 + P2X7RB + 496A increasing by 11.11%, (*p* < 0.0001) (Fig. 2J).

### Effects of P2X7R variants and SNPs on ALP and mineralisation

**Fig. 3:**
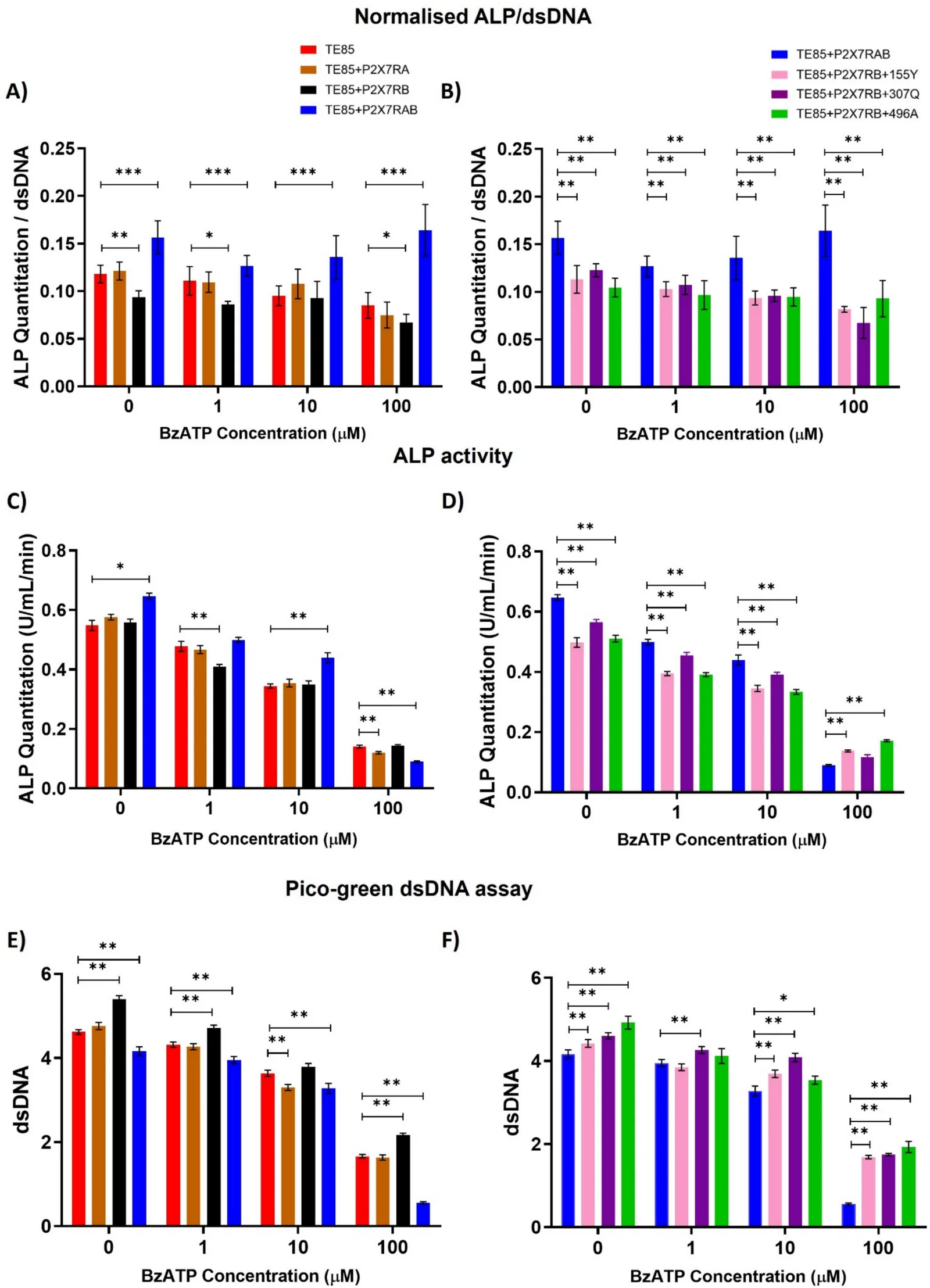
ALP activity of the different TE85 P2X7R variants and SNP cell lines when stimulated with different concentrations of BzATP. All cell variants were seeded into 96-well plates at a density of 1,000 cells per well in 100µL of complete medium with a range of BzATP concentrations. After 3-days of culture, cells were washed with PBS and lysed in nuclease-free water with 1M Tris pH 8.0–8.3, 0.2M MgCl_2_ and 0.2% Triton-X. Cell lysates were obtained after three freeze and thaw cycles. ALP activity was measured by using PnPP as the chromogenic ALP substrate in the presence of Mg2^+^ ions in a buffered solution. The absorbance was read at 405nm and the fluorescence at 480/520nm excitation/emission. **A**&**B**) The ALP activity (U/ml/min) normalised with the dsDNA concentration (µg/ml); which were individually measured by the PnPP assay for 30 min and picogreen DNA assay in the TE85 variant and SNP cell lines respectively. **C**&**D**) Total ALP activity (U/ml/min) measured by PnPP assay for 30 min in the TE85 variant and SNP cell lines respectively **E**&**F**) The total dsDNA concentration in the above samples; measured by pico-green dsDNA assay in the TE85 variant and SNP cell lines respectively. * *p* < 0.05, ** *p* < 0.01, *** *p* < 0.001. Control data shown across panels were derived from the same experiment and are presented separately for clarity and biologically relevant comparisons

ALP is an enzyme produced by osteoblasts which contributes towards mineralisation, it is therefore a biomarker for osteoblast activity [31]. We performed an ALP assay to assess this important aspect of bone physiology when expressing the different P2X7R variants and SNPs, both alone or when treated with varying concentrations of BzATP. The total ALP and DNA assay results revealed that the TE85 + P2X7RB cells had the highest dsDNA content suggesting they grew much faster than the TE85 cells when transfected (*p* < 0.01, Fig. 3F), however, the TE85 + P2X7RB cells didnʼt have a higher level of total ALP activity (Fig. 3C) and was lower than in the TE85 cells. On the contrary, the ALP activity per dsDNA was lower in the TE85 + P2X7RB cells than the original untransfected TE85 cells (*p* < 0.01, Fig. 3A). In contrast, the TE85 + P2X7RAB cells had the lowest dsDNA content but had the highest ALP activity, suggesting they grew the slowest, but had an increased osteoblastic phenotype. TE85 + P2X7RA cells showed no difference in ALP activity (Fig. 3A).

The ALP activity was again highest in the TE85 + P2X7RAB cell line compared to all of the SNP co-transfected cell lines (*p* < 0.01, Fig. 3B, D) although all SNP co-transfected cells had a significantly higher dsDNA content (*p* < 0.01, Fig. 3F), and therefore, although they grew faster consistent with previous P2X7R studies, this did not result in a higher ALP activity. When adding different concentrations of BzATP to stimulate P2X7R function, the cell number generally decreased, with the TE85 + P2X7RAB cells decreasing the most at the 100µM concentration. The normalised ALP activity did not change when increasing BzATP concentration, remaining consistent in both the variant cells (Fig. 3A) and in the SNP cells (Fig. 3B).

**Fig. 4:**
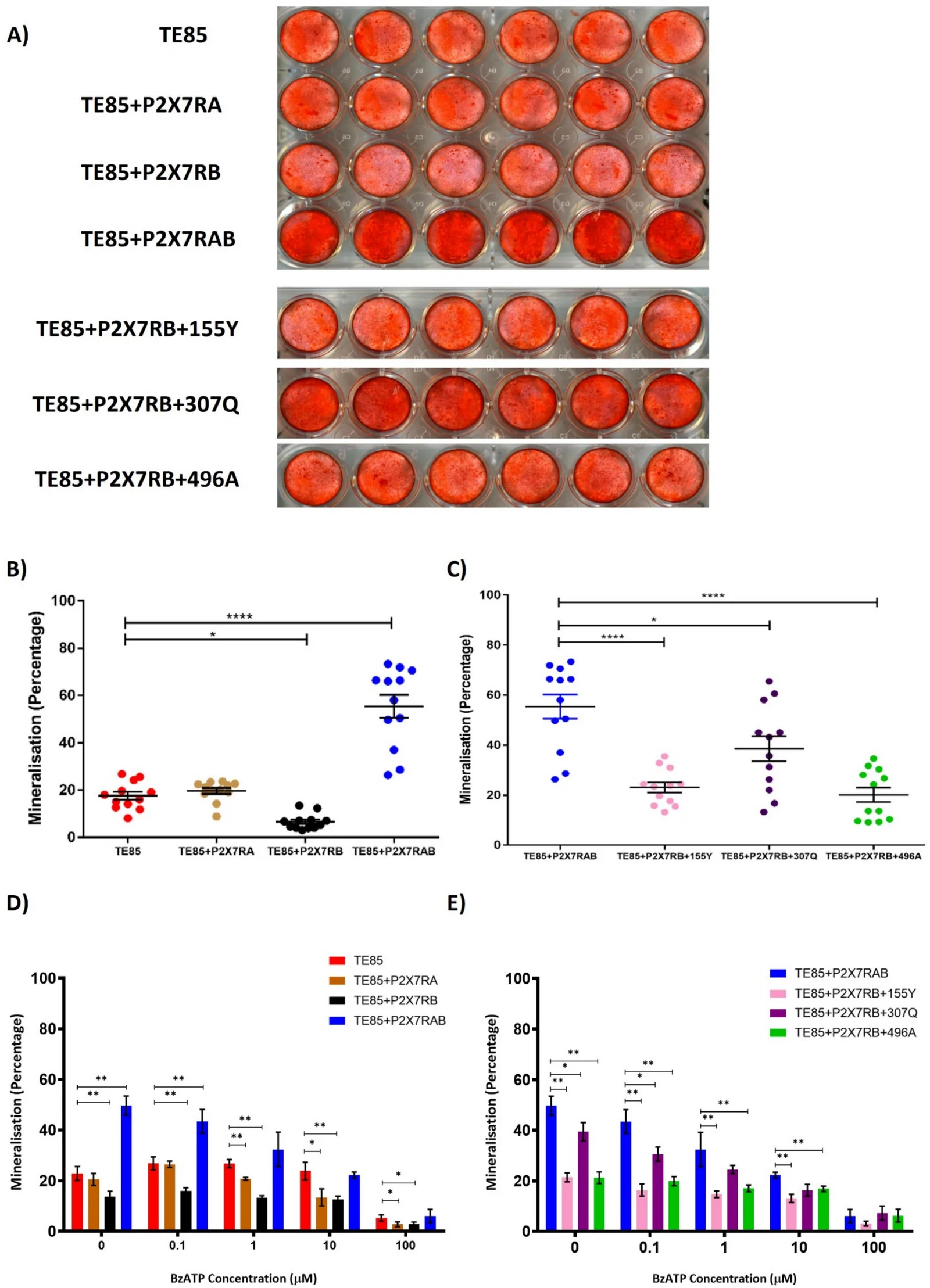
Effects of P2X7R variant and SNPs on osteoblast mineralisation. For mineralisation confluent cells were cultured in osteogenic medium in 24-well plates for 7 days, followed by the addition of inorganic phosphates and Alizarin red staining. **A**) Representative Alizarin red staining with different TE85 variant and SNP cell lines in their well plates. **B**) The quantified percentage of each stained cell line for the different variants, compared to TE85 cells. **C**) The quantified percentage of each stained cell line for the different SNPs compared to TE85 + P2X7RAB cells. **D**&**E**) The quantified percentage of each stained cell line when stimulated with different concentrations of BzATP. * *p* < 0.05, ** *p* < 0.01, *** *p* < 0.001. Control data shown across panels were derived from the same experiment and are presented separately for clarity and biologically relevant comparisons

Mineralisation is an important bone process where minerals such as calcium and phosphate are deposited into bone tissue by osteoblasts, forming a strong dense matrix which provides the skeleton with strength and rigidity [32]. This can be replicated in vitro by culturing the cells with the necessary growth factors to promote bone formation (known as osteogenic medium). We therefore measured mineralisation in all the cell lines over 7 days cultured in osteogenic medium. Alizarin red S staining was used to determine the calcium deposits. Mineralisation was detected in all cell lines but with different levels dependent upon receptor variant and SNP expression (Fig. 4A). Quantification of the staining (Fig. 4B) showed that, TE85 cells had 17.6% mineralisation. TE85 + P2X7RA had a similar percentage with 19.6% mineralisation and no significant difference (*p* = 0.9067), whilst transfection of TE85 + P2X7RB significantly decreased the calcium deposition (*p* = 0.0161) and only had 6.5% mineralisation. The co-transfected TE85 + P2X7RAB cells had significantly higher mineralisation with 55.4% compared to TE85 cells (*p* < 0.0001). All the SNP co-transfected cell lines had significantly lower mineralisation than TE85 + P2X7RAB. For the GOF TE85 + P2X7RB + 155Y the percentage mineralisation was 23.10% (*p* < 0.0001), the LOF SNPs had 38.5% mineralisation for TE85 + P2X7RB + 307Q (*p* = 0.0110) and 20.14% mineralisation for the TE85 + P2X7RB + 496A (*p* = 0.0001) (Fig. 4C). Cells were then cultured in osteogenic medium with different BzATP concentrations to investigate how P2X7R activation and the SNPs would affect mineralisation. Quantification of the amount of mineralisation showed the decreasing trend in calcium deposition with the increasing concentration of BzATP, with 100µM BzATP significantly reducing mineralisation in all cell lines (Fig. 4D). Interestingly TE85 + P2X7RB + 307Q has a trend of increased mineralisation when stimulated with low levels of BzATP (Fig. 4E).

### Changes in osteogenic gene expression in the different variant and SNP cell lines

**Fig. 5:**
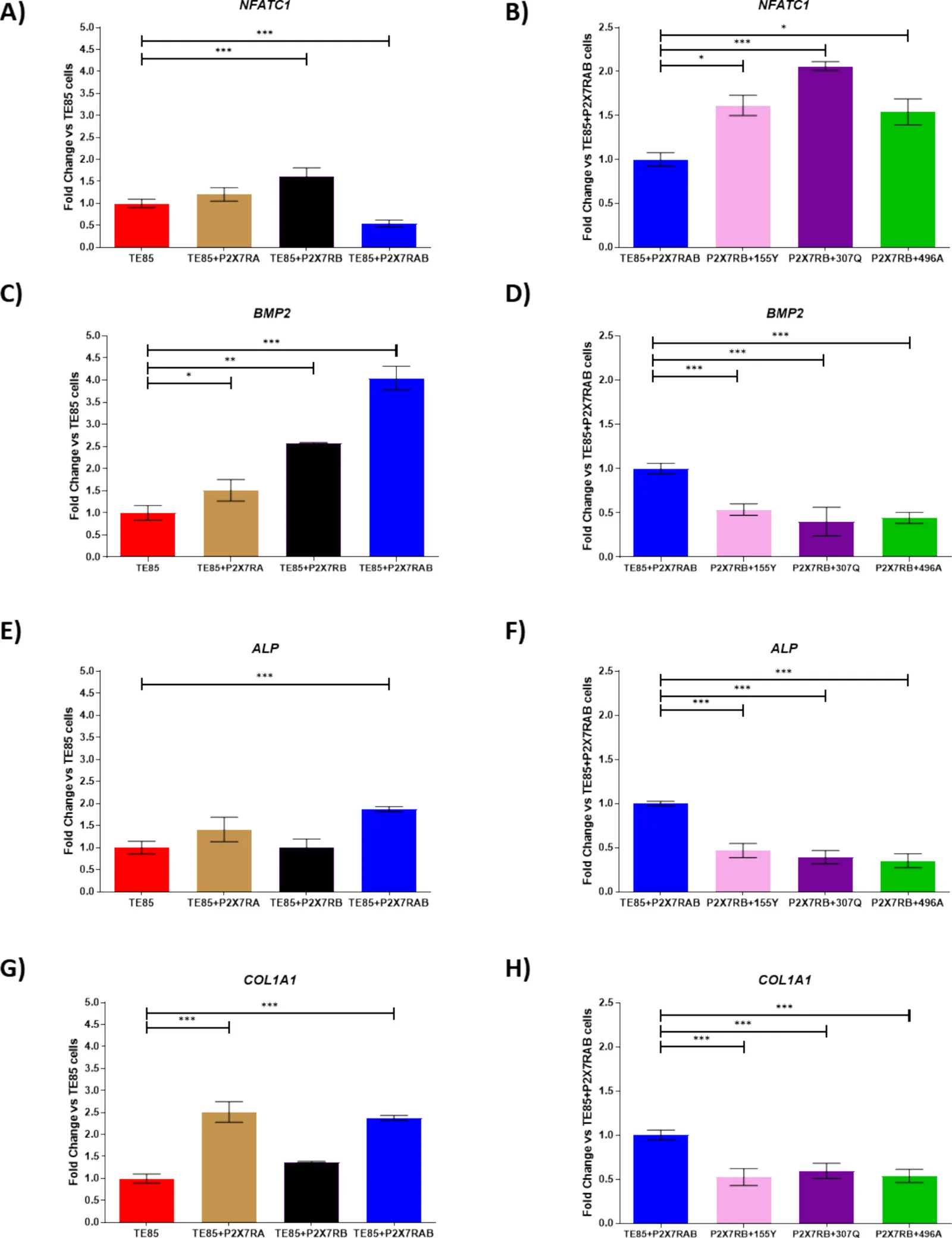
Altered gene expression in the different TE85 variants and SNP expressing cell lines. Various mRNA levels were evaluated by real-time PCR using TaqMan® Assay 384-Well Microfluidic Cards. Expression was normalized to GAPDH endogenous control and is indicated as fold increase over expression levels of TE85 cells for the different variants or TE85 + P2X7RAB for SNP variants. Different genes are shown related to osteogenesis: **A**) *NFATc1* expression in TE85, TE85 + P2X7RA, TE85 + P2X7RB and TE85 + P2X7RAB. **B**) *NFATc1* expression in P2X7R GOF and LOF variants. **C**) *BMP2* expression in TE85, TE85 + P2X7RA, TE85 + P2X7RB and TE85 + P2X7RAB. **D**) *BMP2* expression in P2X7R GOF and LOF variants. **E**) *ALP* expression in TE85, TE85 + P2X7RA, TE85 + P2X7RB and TE85 + P2X7RAB. **F**) *ALP* expression in P2X7R GOF and LOF variants. **G**) *COL1A1* expression in TE85, TE85 + P2X7RA, TE85 + P2X7RB and TE85 + P2X7RAB. **H**) *COL1A1* expression in P2X7R GOF and LOF variants. * *p* < 0.05, ** *p* < 0.01, *** *p* < 0.001. Control data shown across panels were derived from the same experiment and are presented separately for clarity and biologically relevant comparisons

In order to investigate the mechanism associated with the changes in osteoblast phenotype across cell variants and SNPs we used a gene expression array in 384-Well Microfluidic Cards to examine osteogenic gene changes. *NFATc1* activation is associated with regulating bone formation [33] and mass [34] in addition to P2X7R mediated proliferation [11]. We found that the *NFATc1* gene expression level when compared to TE85 cells, was significantly higher in TE85 + P2X7RB cells (1.62-fold increase, *p* < 0.001), TE85 + P2X7RA alone showed no difference, whereas the P2X7RAB had significantly reduced *NFATc1* expression (49% decrease *p* < 0.001) (Fig. 5A). For the SNP expressing TE85 cells *NFATc1* expression was significantly increased in all variants, TE85 + P2X7RB + 155Y had 1.61-fold increase (*p* < 0.05), TE85 + P2X7RB + 307Q had 2.06-fold increase (*p* < 0.001) and TE85 + P2X7RB + 496A had a 1.54-fold increase (*p* < 0.05) compared to TE85 + P2X7RAB (Fig. 5B).

We also assessed *BMP-2* expression, *BMP-2* can cause osteogenic differentiation of mesenchymal cells to bone cells and plays a role in bone homeostasis and remodelling [35, 36]. *BMP-2* was significantly increased in all P2X7R variants, but to varying degrees. TE85 + P2X7RA had a 1.51-fold increase, (*p* < 0.05) TE85 + P2X7RB had a 2.57-fold increase (*p* < 0.01), and TE85 + P2X7RAB had the highest increase of 4.04 fold, (*p* < 0.001) (Fig. 5C). In comparison to TE85 + P2X7RAB the SNP expressing TE85 cells had a significantly decreased *BMP-2* expression in all variants (*p* < 0.001), TE85 + P2X7RB + 155Y had a 47% decrease, TE85 + P2X7RB + 307Q had a 60% decrease and TE85 + P2X7RB + 496A had a 56% decrease (Fig. 5D).

In addition to the previous ALP assay, we examined the expression of the *ALP* gene in these cell isoform variants and SNPs. In line with the previous results, when comparing the isoforms only TE85 + P2X7RAB cells had significantly increased expression compared to TE85 cells (*p* < 0.05), with a 1.87-fold increase (Fig. 5E). The SNP expressing TE85 cells had a significantly decreased *ALP* expression when compared to TE85 + P2X7RAB cells. The TE85 + P2X7RB + 155Y SNP had a 54% decrease (*p* < 0.001) TE85 + P2X7RB + 307Q had a 61% decrease (*p* < 0.01) and TE85 + P2X7RB + 496A had a 65% decrease (*p* < 0.001) (Fig. 5F).

The final gene examined was *COL1A1*, this codes for the alpha 1 chain of type 1 collagen and is a key component of the skeletal system for structural support and strength and is produced by osteoblasts during bone mineralisation. Its function is to act as a scaffold and it is the most prominent protein in bone [37]. *COL1A1* expression was significantly increased in the P2X7RA (2.51-fold increase *p* < 0.001) and P2X7RAB (2.37-fold increase *p* < 0.001) cell lines with no difference for P2X7RB. All SNP expressing TE85 cells had reduced *COL1A1* expression, TE85 + P2X7RB + 155Y SNP had 48% decrease (*p* < 0.001) TE85 + P2X7RB + 307Q had 31% decrease (*p* < 0.01) and TE85 + P2X7RB + 496A had a 46% decrease (*p* < 0.001) in comparison to TE85 + P2X7RAB (Fig. 5G).

## Discussion

Bone-related disorders are often linked to dysregulated bone remodelling processes, where the P2X7R plays a crucial role. The complexity of P2X7R signalling influenced by different isoforms and specific SNPs remains poorly understood. P2X7R has been studied in the context of bone physiology and in bone cancer, however, our study is the first to examine the full length P2X7RA, truncated P2X7RB, co-expression of both isoforms and the effect of different receptor SNPs, demonstrating how these variants interact and impact osteoblast function at the cellular level. Understanding these interactions is crucial for identifying how genetic variations contribute to bone disorders and bone health, this could pave the way for developing targeted therapies that modulate P2X7R activity to restore correct bone remodelling. Our findings provide a new perspective on the role of P2X7R in bone biology on a cellular level, highlighting the receptorʼs potential as a therapeutic target for bone-related diseases.

The SNPs include the c.489C > T variant (p.His155Tyr) GOF, c.946G > A (p.Arg307Gln) LOF and c.1513A > C (p.Glu496Ala) LOF. The cellular osteogenic behaviour of the TE85 cell line was examined for intracellular calcium uptake, pore formation, cell proliferation, ALP activity, mineralisation, and changes in osteogenic gene expression. Having previously demonstrated that the cell line TE85 does not express functional P2X7R [11, 38] we chose to use this cell line to investigate the effect of the splice variants and SNPs by transfecting these cells. We compared the transfected *P2RX7A*, *P2RX7B* and *P2RX7AB* to the original TE85 cells. Due to the SNP variants expressing *P2RX7B* and then the *P2RX7A* variant containing the desired SNP, we compared these to the co-transfected *P2RX7AB*.

We showed that the cell lines expressed the correct variants and SNPs at the mRNA level using end point PCR which we then subsequently sequenced confirming the presence of the SNP (although there was a heterogeneous population for the (p.His155Tyr) GOF). When stimulated with BzATP, calcium concentrations were increased in P2X7RA and P2X7RAB cells however, this was limited in P2X7RB which has previously been shown to have a lower calcium response in other cells such as acute myeloid leukaemia cells [39]. Following this, we found that the co-transfected SNP variant (p.His155Tyr) GOF had a higher calcium response, with the two LOF SNPs reducing the response in line with their expected LOF impact. We additionally checked the BzATP induced pore forming ability of these cell variants. Consistent with our previously published data P2X7RA alone could not form a pore [11] and due to the missing truncated region, neither could the P2X7RB variant. However, co-expression of both variants (P2X7RAB) resulted in pore formation, introduction of both LOF SNPs into the P2X7RAB reduced this ability. As the co-expressed variant usually forms a pore, this suggests the reduction is due to the LOF SNP. The GOF SNP retained the ability to form a functional pore, as well as the observed calcium increase. This is in line with further published literature [18].

This functional data demonstrate that the P2X7RAB variant reproduces a physiologically “healthy” osteogenic signalling profile, with robust calcium influx and pore formation likely underpinning high ALP, mineralisation, and osteogenic gene expression. The LOF SNPs primarily impair these osteogenic outputs, which aligns with clinical observations where p.Arg307Gln and p.Glu496Ala are associated with lower BMD and higher fracture risk [26–28]. In contrast, the GOF SNP p.His155Tyr, despite enhancing calcium signalling and maintaining pore function, did not strongly augment osteogenic differentiation or mineralisation. This may reflect the dual-nature of P2X7R signalling, heterogeneous expression in our transfected cell populations, or the absence of osteoclast interactions that are essential for fully translating GOF effects into anabolic bone outcomes.

Genetic variations that alter P2X7R calcium and pore function are likely to disrupt the delicate balance between bone resorption and formation required to maintain skeletal health. Previous association studies have shown that LOF variants, such as p.Glu496Ala and p.Arg307Gln, are associated with reduced BMD and increased fracture risk in postmenopausal women [26, 27]. Our results show that LOF variants reduce the bone-forming phenotype at the cellular level, suggesting a direct mechanism by which these SNPs contribute to skeletal fragility in vivo, while GOF variants, such as p.Ala348Thr and p.Gln460Arg are shown to be protective [28, 30]. Similarly, stress fracture susceptibility is increased by LOF variants and reduced by GOF variants in active populations [29]. These observations support the idea that the functional impact of P2X7R LOF variants observed in our cell-based experiments may directly relate to bone homeostasis in vivo. Additionally, previous studies have shown that P2X7R interacts with cytoskeletal proteins involved in mechanotransduction and P2X7R knockout mice exhibit a disuse phenotype along with a diminished response to mechanical loading [27] Considering that mechanical loading represents the most potent anabolic stimulus for bone, and that exercise becomes less effective after reaching peak bone mass, early identification of individuals carrying polymorphisms that significantly reduce P2X7R function could be advantageous [27].

Having previously shown that transfection of *P2RX7A*, *P2RX7B* and *P2RX7AB* can provide a growth advantage to TE85 cells [11, 24], we examined this in the SNP variants. All SNP variants increased cell growth compared with the P2X7RAB TE85 cells regardless of SNP, this could be due to the opposing dual function of P2X7R signalling such as through pore formation and calcium signalling. Increased extracellular ATP response and increased calcium signalling resulting from GOF mutations can trigger a number of signalling pathways that encourage cell growth, whereas pore formation may be hampered or rendered ineffective by LOF SNPs, therefore meaning the cells cannot undergo cell death and therefore survive longer. Previous studies further support that non-functional variants of P2X7Rs are linked to increased cell growth [24, 40, 41]. Overall, the apparent paradox of increased proliferation in vitro caused by both GOF and LOF SNP mutations in the *P2RX7* could be potentially explained by the triggering of compensatory mechanisms in both instances as they may trigger cellular pathways that enhance proliferation as a response to disrupted signalling, attempting to balance the bone remodelling process and maintain bone homeostasis. Future studies to determine these complex interactions are needed and may not reflect patient cohort observations. This study is the first indication of the role P2X7RB plays in combination with a GOF SNP that may cooperate in regulating receptor activity in osteoblasts.

When considering the different effects on the bone phenotype with the mineralisation assay, ALP activity and gene expression the full length pore forming P2X7R has the ability to promote osteogenic differentiation from MSCs through shock wave induced ATP release [42], further to this it promotes osteogenic differentiation in postmenopausal bone marrow derived MSCs cells through the activation of protein kinases and cytoskeletal rearrangements [43].

The P2X7RAB variant in our study has this phenotype, it had the highest mineralisation and ALP activity, with high levels of expression of *BMP2*, *ALP*, and *COL1A1*. In contrast to this, the highly proliferative variants such as the single P2X7RA, P2X7RB, or co-transfected SNP variants had reduced mineralisation, reduced ALP and higher levels of *NFATc1* expression. This suggests that disruption of normal P2X7R signalling may alter the balance between osteoblast proliferation and differentiation, promoting a more proliferative but less mature osteoblast phenotype. The increased NFATc1 expression observed in the highly proliferative SNP variants may contribute mechanistically to this shift, as NFAT signalling has previously been associated with regulation of osteoblast proliferation and maturation. In contrast, the fully functional P2X7RAB variant demonstrated a more differentiated osteogenic phenotype characterised by increased matrix-associated gene expression and mineralisation. Therefore, in conclusion our study reveals the complex influence of SNPs and P2X7R isoform variants on osteoblasts, demonstrating two distinct different phenotypes, a less differentiated highly proliferative phenotype when P2X7R is disrupted and a mature bone variant phenotype when the fully functional pore forming variant is present. This could be expanded by looking into other bone markers such as tartrate-resistant acid phosphatase (TRAP), osteopontin (OPN) or RUNX2 [44]. A limitation of the present study is that direct protein-level confirmation of the individual isoforms was not performed and the study relied on functional assessments, however, our data provide a mechanistic bridge between the cellular impact of *P2RX7* SNPs and human clinical outcomes: LOF SNPs impair osteoblast differentiation, consistent with bone loss and reduced BMD seen in patient cohorts, whereas GOF SNPs do not strongly enhance osteogenesis in vitro, reflecting the complexity of P2X7Rʼs dual signalling roles and the need for systemic interactions. This underscores that the effects of P2X7R variants cannot be interpreted solely based on calcium/pore function but must be considered in the broader physiological context including osteoclast activity, bone microenvironment and mechanical loading [26–30, 45]. The gene expression revealed alterations in osteogenic genes, but more research such as functional genomics or pathway analyses, could be used to reveal the molecular processes underlying the alterations. The clinical implications of our findings should also be taken into account in future research. Investigating links between any *P2RX7* genetic variations and bone-related diseases may pave the way for personalised medicine approaches to bone health management and could identify associations with increased/reduced BMD or accelerated bone loss or fracture risk in a number of bone-related pathologic conditions.

## Summary

In summary, our study demonstrates that the P2RX7AB variant promotes a mature, differentiated osteogenic phenotype, with robust calcium signalling, pore formation, high ALP activity, mineralisation, and osteogenic gene expression. In contrast, cells expressing only P2X7RA, P2X7RB, or P2X7RAB with LOF SNPs adopt a more proliferative, less differentiated phenotype, showing reduced mineralisation and osteogenic gene expression, consistent with bone loss phenotypes observed clinically. The GOF SNP provides calcium and pore responses but does not strongly increase osteogenic differentiation, likely due to the dual nature of P2X7R signalling, heterogeneous cell populations and a lack of osteoclast and other bone microenvironment interactions. This study provides the first evidence that the P2X7RB isoform can interact with the 155Y GOF variant of P2X7RA, suggesting it may cooperate in regulating receptor activity in osteoblasts. These findings link *P2RX7* genetic variations to distinct osteoblast phenotypes and provide mechanistic insight into how different SNPs influence skeletal health, highlighting P2X7R as a potential target for modulating bone remodelling.

## Supplementary Information

Below is the link to the electronic supplementary material.

Supplementary Material 1 (DOCX 14.8 KB)

## Data Availability

No datasets were generated or analysed during the current study.
