# Supplementary material for: The effect of P2RX7 functional SNPs on osteoblast cell phenotype, function and signalling- roles in bone homeostasis and osteogenesis
Source: Purinergic Signal. 2026 Jun 27;22(4):61. doi: 10.1007/s11302-026-10171-5 (PMC13310211; doi:10.1007/s11302-026-10171-5)
Supplement: Supplementary file 1 — Supplementary Material 1 (DOCX 14.8 KB) [file 11302_2026_10171_MOESM1_ESM.docx]

**Supplementary**

**Supplementary Table 1: List of genes in the 384-Well Microfluidic Cards**

|  | **Gene name** | **Gene Id** | **Function** |
| --- | --- | --- | --- |
| 1 | *ALP* | Hs01029144_m1 | Osteoblast differentiation |
| 2 | *BMP2* | Hs00154192_m1 | Osteoblast differentiation |
| 3 | *BMP4* | Hs03676628_s1 | Osteoblast differentiation |
| 4 | *BMP7* | Hs00233476_m1 | Osteoblast differentiation |
| 5 | *CBFA1/RUNX2* | Hs00231692_m1 | Osteoblast differentiation/function |
| 6 | *Osteocalcin* | Hs01587814_g1 | Mineralisation |
| 7 | *Osteopontin* | Hs00959010_m1 | Mineralisation |
| 8 | *C-FOS* | Hs04194186_s1 | Cell survival/proliferation |
| 9 | *MCSF* | Hs00174164_m1 | Osteoclast regulator |
| 10 | *RANKL* | Hs00243522_m1 | Osteoclast regulator |
| 11 | *OPG* | Hs00900358_m1 | Osteoclast regulator |
| 12 | *COL1A1* | Hs00164004_m1 | Collagen type 1, alpha 1 |
| 13 | *NFATc1* | Hs00542678_m1 | Proliferation |
| 14 | *P2RX7* | Hs00175721_m1 | P2RX7 gene |
| 15 | *Beta-catenin* | Hs00355049_m1 | Wnt-signalling |
| 16 | *SFRP-1* | Hs00610060_m1 | Wnt antagonist |
| 17 | *Caspase-3* | Hs00234387_m1 | Apoptosis related |
| 18 | *Caspase-9* | Hs00154261_m1 | Apoptosis related |
| 19 | *BAX* | Hs00180269_m1 | Apoptosis related |
| 20 | *BCL-2* | Hs00608023_m1 | Apoptosis related |
| 21 | *18S* | Hs99999901_s1 | Housekeeping |
| 22 | *GAPDH* | Hs02758991_g1 | Housekeeping |
| 23 | *HPRT1* | Hs02800695_m1 | Housekeeping |
| 24 | *B-actin* | Hs01060665_g1 | Housekeeping |
